# Does subclinical hypothyroidism affect the prognosis of patients with chronic systolic heart failure: A systematic review and meta-analysis

**DOI:** 10.1097/MD.0000000000038410

**Published:** 2024-06-07

**Authors:** Xiao Han, Xiuge Wang

**Affiliations:** a Department of Endocrinology and Metabolism, Changchun University of Traditional Chinese Medicine, Changchun, Jilin Province, China; b Department of Endocrinology and Metabolism, Affiliated Hospital of Changchun University of Traditional Chinese Medicine, Changchun, Jilin Province, China.

**Keywords:** chronic systolic heart failure, meta-analysis, prognosis, subclinical hypothyroidism

## Abstract

**Background:**

Chronic systolic heart failure (CSHF) is a significant health burden with high morbidity and mortality. The role of subclinical hypothyroidism (SCH) in the prognosis of CSHF patients remains a critical area of inquiry. This systematic review and meta-analysis aim to elucidate the impact of SCH on the prognosis of patients with CSHF.

**Methods:**

Adhering to Preferred Reporting Items for Systematic Reviews and Meta-Analyses guidelines, this meta-analysis employed a comprehensive search strategy across major databases including PubMed, Embase, Web of Science, and the Cochrane Library. The Patient, Intervention, Comparison, Outcome framework guided the inclusion of studies focusing on patients with CSHF, comparing those with and without SCH. Quality assessment was performed using the Newcastle–Ottawa scale. Statistical analyses assessed heterogeneity and publication bias, employing fixed-effect or random-effects models based on heterogeneity levels.

**Results:**

From an initial pool of 1439 articles, 8 studies met the stringent inclusion criteria. These studies, conducted across diverse geographical regions, highlighted the relationship between SCH and all-cause mortality, cardiac events, and subgroup differences in CSHF patients. The meta-analysis revealed SCH as a significant risk factor for all-cause mortality (HR = 1.42) and cardiac events (HR = 1.46). Subgroup analysis indicated variability in risk based on region, sample size, age, and follow-up duration. Sensitivity analysis confirmed the stability of these findings, and publication bias assessment indicated symmetric funnel plot and nonsignificant Egger test results.

**Conclusions:**

SCH emerges as a predictive factor for all-cause mortality, cardiovascular events, and rehospitalization in CSHF patients. This finding underscores the importance of screening for SCH in CSHF patients, highlighting its potential role in improving patient prognosis.

## 1. Introduction

Chronic systolic heart failure (CSHF), a critical and prevalent cardiovascular condition, represents a significant global health burden due to its high morbidity and mortality rates. It is characterized by the heart’s diminished capacity to pump blood effectively, leading to a variety of clinical symptoms and a progressive decline in cardiac function.^[1,2]^ The etiology of CSHF is multifactorial, encompassing a spectrum of pathophysiological changes that compromise cardiac output and contribute to the clinical syndrome of heart failure. The deleterious impact of CSHF on public health is substantial. Patients with CSHF often experience a decline in their quality of life due to physical limitations, recurrent hospitalizations, and the psychological burden of a chronic illness.^[3]^ This condition not only imposes a significant economic burden on healthcare systems worldwide due to the cost of long-term management but also has profound social implications, affecting patients and their families.^[4]^

In recent years, subclinical hypothyroidism (SCH), a condition characterized by elevated thyroid-stimulating hormone (TSH) levels with normal free thyroxine (T4) levels, has gained attention in the context of CSHF.^[5]^ SCH, often asymptomatic, is increasingly recognized as a potential contributor to cardiovascular morbidity. The thyroid hormone plays a critical role in cardiovascular homeostasis, influencing heart rate, myocardial contractility, vascular resistance, and blood pressure. Consequently, even subtle alterations in thyroid function, such as those seen in SCH, may have significant implications for patients with CSHF. Emerging evidence suggests that SCH may adversely affect cardiac function and structure, potentially exacerbating heart failure symptoms and influencing the clinical course of CSHF.^[6,7]^ However, the extent to which SCH impacts the prognosis of patients with CSHF remains a subject of considerable debate. This ambiguity underscores the need for a comprehensive systematic review and meta-analysis to elucidate the relationship between SCH and CSHF.

The objective of this study is to critically analyze existing literature on the impact of SCH on the prognosis of patients with CSHF. By synthesizing data from various studies, this systematic review and meta-analysis aim to provide a clearer understanding of how SCH influences the clinical outcomes of CSHF.

## 2. Materials and methods

### 2.1. Search strategy

In conducting our meta-analysis, we employed a search strategy that adhered strictly to the Preferred Reporting Items for Systematic Reviews and Meta-Analyses (PRISMA) guidelines.^[8]^ The meta-analysis is structured around the Patient, Intervention, Comparison, Outcome (PICO) framework, elucidating the following aspects: Patient (P): this study focused on patients diagnosed with CSHF. Intervention (I): the intervention in consideration was the presence of SCH. Comparison (C): the comparison was made between CSHF patients with SCH and those without SCH. Outcome (O): the outcome of interest was the prognosis of CSHF, including changes in cardiac function, symptom severity, and overall survival rates.

We conducted a comprehensive search across 4 major electronic databases – PubMed, Embase, Web of Science, and Cochrane Library – on September 19, 2023, without time constraints. We employed specific search terms aligned with the PICO components: “heart failure,” “cardiac failure,” “SCH,” and “prognosis.” These terms were carefully selected to ensure a comprehensive review of the pertinent literature. Our search strategies for each database are detailed in Supplementary S1, Supplemental Digital Content, http://links.lww.com/MD/M762, demonstrating our method’s transparency and reproducibility. Furthermore, language restrictions were not imposed, and we manually reviewed reference lists to identify additional relevant studies.

### 2.2. Inclusion criteria and exclusion criteria

#### 2.2.1. Inclusion criteria

1. *Study design.* Included studies were randomized controlled trials (RCTs), cohort studies, case–control studies, and cross-sectional studies.
2. *Participants.* The studies included patients diagnosed with CSHF.
3. *Intervention.* The primary intervention examined was the presence of SCH.
4. *Comparators.* Comparisons were made between patients with CSHF with SCH and those without SCH.
5. *Outcomes.* Studies were included if they reported on the prognosis of CSHF, particularly focusing on changes in cardiac function, symptom severity, and overall survival rates.

#### 2.2.2. Exclusion criteria

1. *Nonrelevant studies.* Studies not specifically addressing the prognosis of CSHF in the context of SCH were excluded.
2. *Study quality.* Studies with insufficient data, poor methodology, or lack of peer-review were excluded.
3. *Duplicate publications.* Studies that were duplicate publications or subsets of larger studies already included in the analysis were excluded.
4. *Animal studies.* Studies conducted on animals or in vitro studies were excluded.
5. *Case reports and editorials:* Case reports, editorials, and opinion pieces without original data were excluded.

### 2.3. Data extraction

In the execution of this meta-analysis, the process of literature screening and data extraction was meticulously conducted. This task was independently carried out by 2 evaluators, who subsequently cross-checked their findings to ensure accuracy and consistency. During the data extraction phase, in instances where discrepancies arose, the involved reviewers engaged in discussion to resolve these issues. If a consensus could not be reached, a 3rd-party reviewer was consulted for an objective resolution. The extracted data encompassed several key elements: the authors of each study, the year of publication, the geographical region where the study was conducted, the number of participants involved in each study, the specific definition of SCH employed, the duration of follow-up, and the average age of the study participants. Furthermore, in situations where the published reports lacked specific data of interest, efforts were made to contact the investigators of the original studies. These communications were primarily conducted via email, with the aim of requesting access to any relevant unpublished data that could contribute to the comprehensiveness and depth of our meta-analysis.

### 2.4. Quality assessment

In our meta-analysis, the evaluation of study quality was a critical step, undertaken with meticulous care. This assessment was conducted by 2 independent reviewers who utilized the Newcastle–Ottawa scale (NOS) for this purpose.^[9]^ The NOS, renowned for its efficacy in research evaluation, assesses study quality across 3 vital domains: selection of study groups, comparability of groups, and ascertainment of either the exposure or outcome of interest for case-control or cohort studies, respectively. This comprehensive assessment allowed for a detailed examination of potential biases present within the studies. Based on the NOS criteria, each study received a score out of a maximum of 9 points, providing a quantitative measure of its quality. The scoring interpretation was structured as follows: studies with a total score ranging from 0 to 3 were categorized as low quality, indicating significant methodological limitations. Studies scoring between 4 and 6 were considered of moderate quality, suggesting a reasonable level of research reliability. Finally, studies achieving a score between 7 and 9 were classified as high quality, reflecting robust methodological standards and a high degree of reliability in their findings.

### 2.5. Statistical analyses

The statistical approach adopted in our meta-analysis was comprehensive and adhered to standardized protocols. We initiated our analysis by assessing the heterogeneity between studies. This was accomplished using chi-square statistics, with the degree of heterogeneity quantified by the *I*^2^ value. The overall effect size was assessed by the hazard ratio (HR) with a 95% confidence interval (CI), providing a measure of the impact of SCH on the prognosis of patients with CSHF. In instances where the *I*^2^ value fell below 50%, coupled with a corresponding *P* value of .10 or higher, heterogeneity was deemed insignificant. Under these conditions, the fixed-effect model was employed to compute the combined effect size, reflecting a more homogenous set of studies. Conversely, significant heterogeneity was identified when the *I*^2^ value was 50% or greater, or the corresponding *P* value was <.10. In such cases, the random-effects model was utilized, acknowledging the variability across studies. The use of this model allowed for a broader inference, accounting for the potential differences between the studies. Furthermore, to detect any potential publication bias, the symmetry of the funnel plot was examined. An equitable distribution of study results on either side of the funnel plot’s apex would indicate a reduced risk of publication bias. Additionally, Egger linear regression test was applied as a quantitative tool for the detection of publication bias. This method provided a more rigorous statistical assessment of the likelihood of bias influencing the meta-analysis outcomes. All statistical tests conducted were 2-sided, with a *P* value threshold of <.05 set for statistical significance. The data analysis was performed using Stata version 17 (StataCorp, College Station, TX, USA), ensuring a high standard of analytical rigor and accuracy.

## 3. Results

### 3.1. Search results and study selection

**Figure 1.**
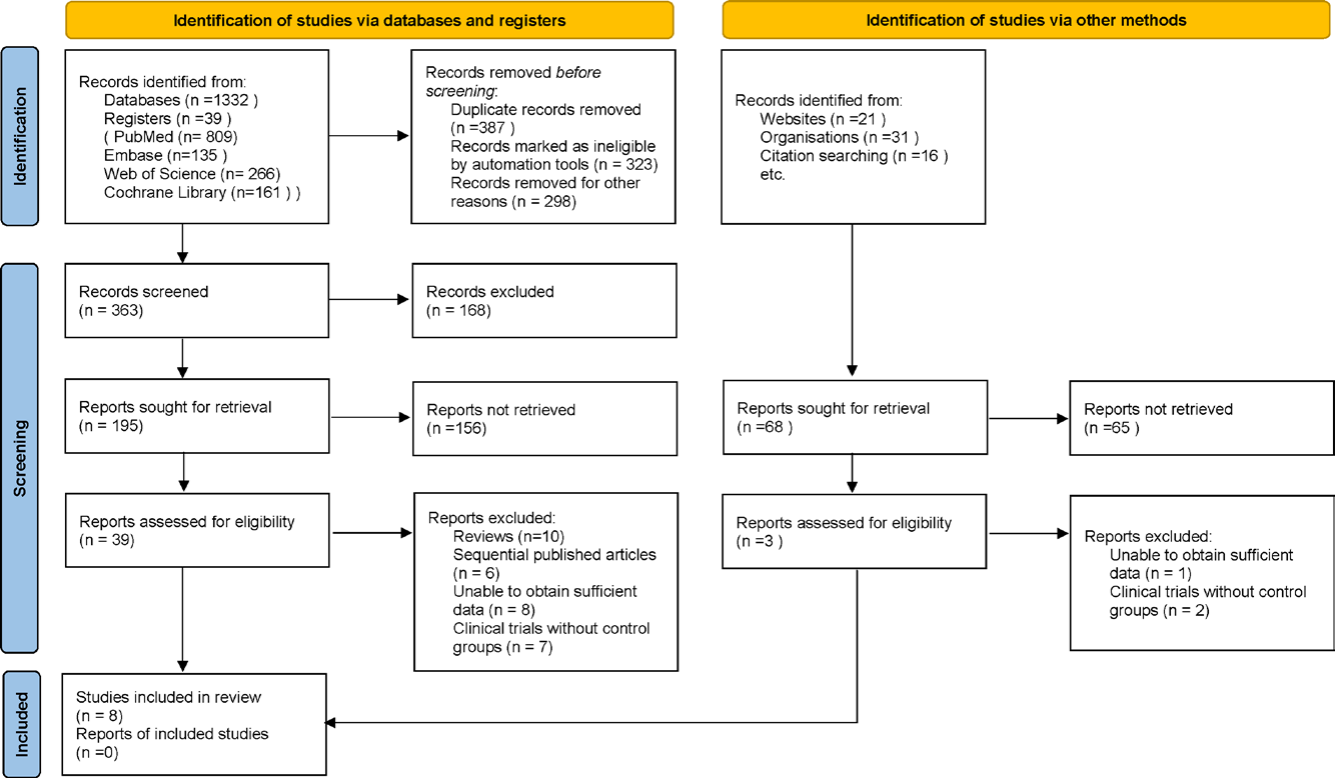
Flowchart depicting the study selection process for inclusion in the meta-analysis.

Search results and study selection process meticulously adhered to predefined criteria, ensuring methodological rigor. Initially, a total of 1439 articles were identified across multiple databases and other methods. The identification process through databases and registers yielded 1371 records. Following the removal of 387 duplicates, 323 records through automation tools for ineligibility, and 298 for various other reasons, 168 records were excluded. Additionally, 156 reports were not retrieved, leading to 39 reports assessed for eligibility. Out of these, 10 were excluded as review articles, 6 due to sequential publication, 8 for insufficient data, and 7 clinical trials lacking control groups, resulting in 8 studies included from this method. Concurrently, an alternative search via other methods identified 68 records, with 65 reports not retrieved and 3 excluded (one for insufficient data and 2 clinical trials without control groups), contributing no additional studies to the final inclusion. Ultimately, this rigorous and transparent process culminated in 8 studies meeting the stringent inclusion criteria for our meta-analysis^[10–17]^ (Fig. 1).

### 3.2. Study characteristics

The meta-analysis encompassed a diverse range of studies, characterized by varied geographic locations and participant demographics. The studies were conducted between 2008 and 2018, with a representation from multiple regions including Germany, Italy, the USA, China, Europe, and Japan. The mean age of participants across these studies ranged from 51 to 75.3 years, reflecting a wide demographic spread. The sample sizes varied significantly, ranging from 353 to 5046 participants in each study, with a subset of these populations diagnosed with SCH. This variance in sample sizes underscores the extensive scope of the analysis. The definition of SCH across these studies was based on varying thresholds of TSH levels, with cutoffs ranging from 4.0 to 5.5 mU/L, indicating differing clinical approaches to diagnosing SCH. Moreover, the duration of follow-up in these studies ranged from 14.3 to 50 months, providing a comprehensive temporal perspective on the progression and outcomes of CSHF in the context of SCH (Table 1).

**Table 1:** Basic characteristics of included studies.

First author	Yr	Mean age (yrs)	Mean follow-up duration (mo)	Sample size (euthyroid/subclinical hypothyroidism)	Region	Definition of subclinical hypothyroidism
Frey et al^[10]^	2013	68	37	628/34	Germany	TSH > 4.0 mU/L
Iacoviello et al^[11]^	2008	64	15	303/34	Italy	TSH > 5.5 mU/L
Kannan et al^[12]^	2018	56.6	50	1006/74	USA	TSH > 4.51mU/L
Li et al^[13]^	2014	52.1	42	816/79	China	TSH > 5.5 mU/L
Nanchen et al^[14]^	2012	75.3	38.4	5046/199	Europe	TSH > 4.5 mU/L
Rhee et al^[15]^	2013	52.3	14.3	410/54	USA	TSH > 4.7 mU/L
Sato et al^[16]^	2018	68	36.6	911/132	Japan	TSH > 4.0 mU/L
Wang et al^[17]^	2015	51	17	353/41	China	TSH > 4.78 mU/L

TSH = thyroid-stimulating hormone.

### 3.3. Results of quality assessment

The methodological quality of the studies included in the meta-analysis was rigorously evaluated using the NOS. This assessment provided a comprehensive understanding of the methodological rigor inherent in each study. The results revealed a generally high quality among the included studies: 2 studies achieved a score of 7 points, indicating good methodological standards, while 3 studies scored 8 points and another 3 scored 9 points, reflecting very high methodological quality. Notably, none of the studies implemented blinding, and there was an absence of allocation concealment across all studies. The overall risks of bias and their corresponding ratios were carefully compiled and are detailed in Table 2.

**Table 2:** The quality assessment according to Newcastle–Ottawa Scale of each cohort study.

Study	Selection				Comparability	Outcome			Total score
	Representativeness of the exposed cohort	Selection of the nonexposed cohort	Ascertainment of exposure	Demonstration that outcome	Comparability of cohorts	Assessment of outcome	Was follow-up long enough	Adequacy of follow-up of cohorts	
Frey et al^[10]^	★	★	★	★	★★	★	★	★	9
Iacoviello et al^[11]^		★	★	★	★★	★	★	★	8
Kannan et al^[12]^	★	★		★	★	★	★	★	7
Li et al^[13]^	★	★	★	★	★★	★		★	8
Nanchen et al^[14]^	★	★	★	★	★★	★	★	★	9
Rhee et al^[15]^	★	★	★	★	★	★	★	★	8
Sato et al^[16]^	★	★	★	★	★★	★	★	★	9
Wang et al^[17]^	★	★		★	★	★	★	★	7

### 3.4. Impact of SCH on all-cause mortality

**Figure 2.**
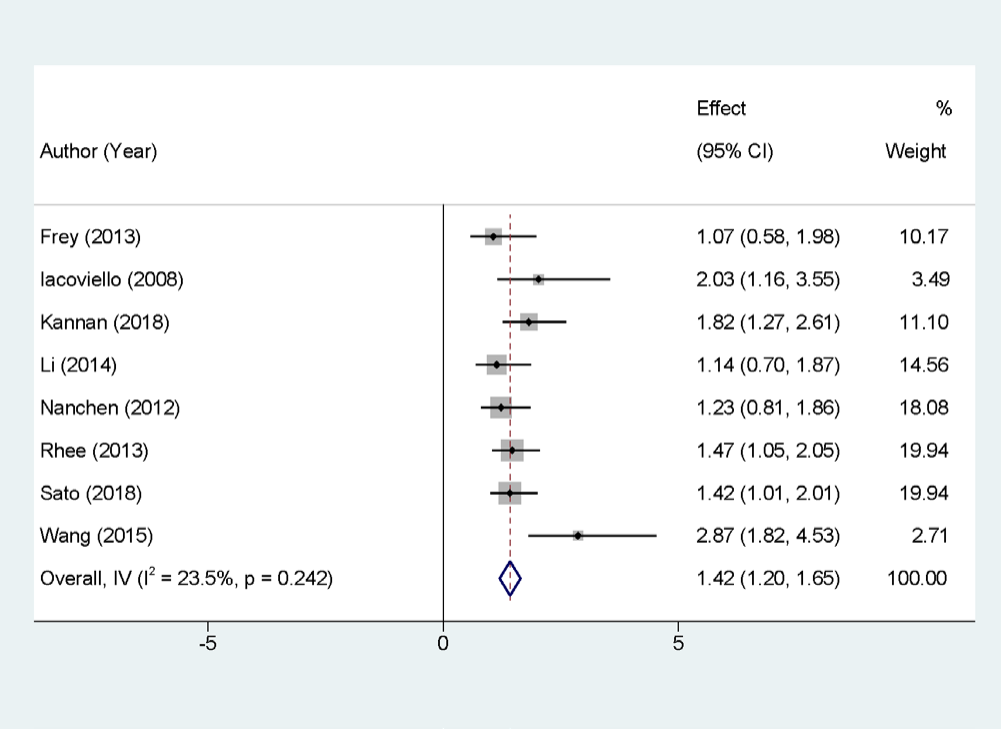
Forest plot illustrating the relationship between subclinical hypothyroidism and all-cause mortality in patients with chronic systolic heart failure.

Our meta-analysis synthesized data from 8 studies that examined the effect of SCH on all-cause mortality in patients with heart failure. The pooled analysis revealed no significant statistical heterogeneity among the study results (*P* = .242, *I*^2^ = 23.5%). Employing a fixed-effect model for the meta-analysis, the findings indicated that SCH is a risk factor for increased all-cause mortality in heart failure patients. Specifically, the HR was 1.42 with a 95% CI of 1.20 to 1.65, and the result was statistically significant (*P* < .05). This outcome is graphically represented in Figure 2.

### 3.5. Influence of SCH on cardiac events

**Figure 3.**
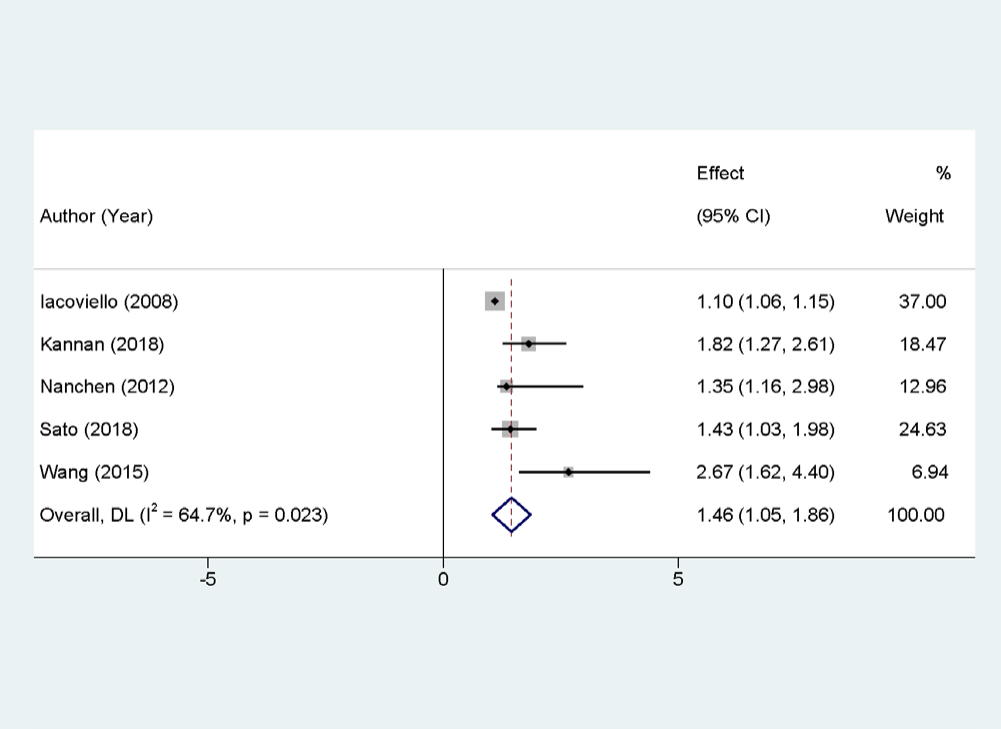
Forest plot presenting the effect of subclinical hypothyroidism on the incidence of cardiac events.

In our meta-analysis, data from 5 studies were collated to assess the impact of SCH on the incidence of cardiac events. A significant level of statistical heterogeneity was observed among these studies (*P* = .023, *I*^2^ = 64.7%), necessitating the use of a random-effects model for the analysis. The results indicated that SCH poses a notable risk factor for cardiac events, with an HR of 1.46 and a 95% CI ranging from 1.05 to 1.86, affirming statistical significance (*P* < .05). This relationship is visually depicted in Figure 3.

### 3.6. Subgroup analysis of SCH and all-cause mortality

This meta-analysis conducted a subgroup analysis to explore the association between SCH and all-cause mortality among heart failure patients (Table 3). The results were stratified by region, sample size, age, and follow-up duration. In terms of region, the HR for Asia was 1.39 (95% CI: 1.10–1.75) with no heterogeneity (*I*^2^ = 0%), and for Europe & America, the HR was 1.44 (95% CI: 1.00–2.10) with moderate heterogeneity (*I*^2^ = 62%). Regarding sample size, smaller studies (<500 participants) showed a higher risk (HR = 1.63, 95% CI: 1.10–2.50) with high heterogeneity (*I*^2^ = 83%), while larger studies (≥500 participants) reported an HR of 1.23 (95% CI: 0.99–1.55) with no observed heterogeneity. Age-wise, the risk was higher in the ≤65 years group (HR = 1.68, 95% CI: 1.15–2.50) with significant heterogeneity (*I*^2^ = 66%), compared to the >65 years group (HR = 1.18, 95% CI: 1.02–1.35) which showed no heterogeneity. Finally, for follow-up duration, studies with less than 2 years of follow-up reported an HR of 1.92 (95% CI: 1.30–2.95) with substantial heterogeneity (*I*^2^ = 62%), while studies with longer follow-up showed an HR of 1.17 (95% CI: 1.02–1.34) with no heterogeneity.

**Table 3:** Subgroup analysis of the association between subclinical hypothyroidism and all-cause mortality.

Subgroup	HR (95% CI)	*I *^2^ (%)	*P* value for effect size
Region			
Asia	1.39 (1.10–1.75)	0	.4
Europe and America	1.44 (1.00–2.10)	62	.02
Sample size			
<500	1.63 (1.10–2.50)	83	.005
≥500	1.23 (0.99–1.55)	0	.82
Age (yrs)			
≤65	1.68 (1.15–2.50)	66	.04
>65	1.18 (1.02–1.35)	0	.72
Follow-up duration (Yrs)			
<2	1.92 (1.30–2.95)	62	.08
≥2	1.17 (1.02–1.34)	0	.85

CI = confidence interval, HR = hazard ratio.

### 3.7. Sensitivity analysis: assessing the impact of SCH on cardiac events

**Figure 4.**
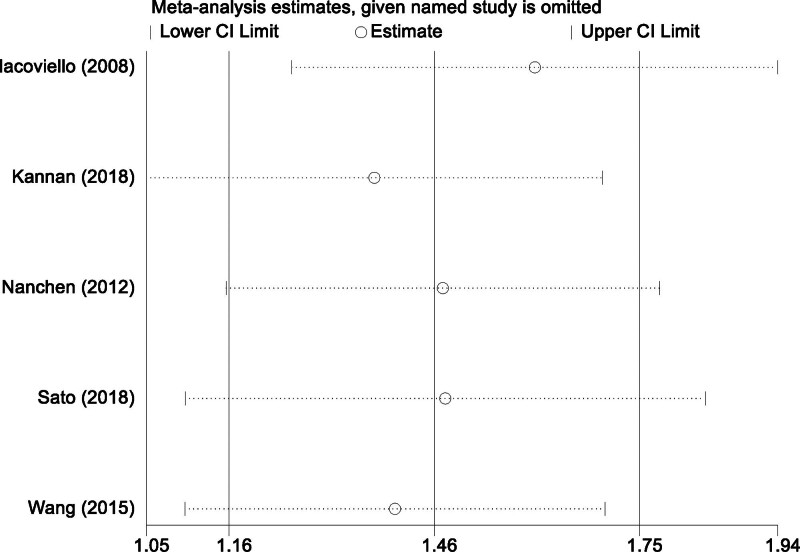
Graph demonstrating the results of the sensitivity analysis to assess the stability of findings regarding subclinical hypothyroidism’s impact.

In light of the considerable heterogeneity identified in our meta-analysis regarding the impact of SCH on cardiac events, we implemented a sensitivity analysis to validate the robustness and consistency of our findings. This analysis was pivotal in determining the influence of each individual study on the collective outcome. The process involved systematically removing 1 study at a time from the pool and recalculating the overall effect size based on the remaining studies. This step-by-step exclusion approach was crucial in identifying any single study that might disproportionately skew the overall results. The outcome of this sensitivity analysis was revealing. Despite the sequential omission of each study, the pooled effect size remained largely unaffected, demonstrating a high degree of stability and consistency in our results. This outcome suggests that the overall conclusions of our meta-analysis are not overly reliant on any single study, thereby bolstering the credibility and reliability of our conclusions (Fig. 4).

### 3.8. Publication bias assessment in meta-analysis

**Figure 5.**
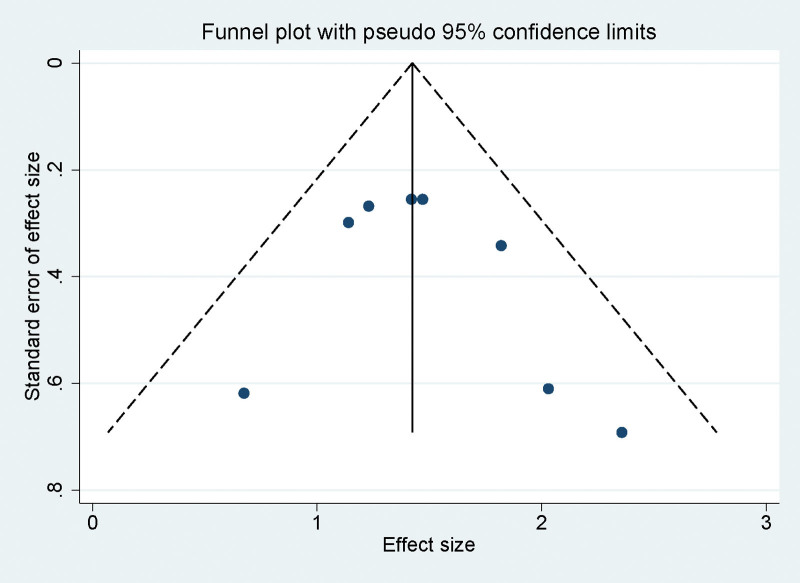
Funnel plot analyzing publication bias across all studies included in the meta-analysis.

To evaluate the presence of publication bias within our meta-analysis, we employed both visual and statistical methods. The symmetry of the funnel plot, which encompassed all included studies, was carefully examined. These plots, as depicted in Figure 5, displayed a symmetric distribution, suggesting an absence of significant publication bias. Furthermore, to substantiate these visual findings, Egger linear regression test was utilized. This statistical approach is specifically designed to detect bias in meta-analyses. The test results for different variables in our meta-analysis uniformly yielded *P* values > .05. This uniformity across various parameters indicates a lack of significant publication bias.

## 4. Discussion

CSHF remains a formidable challenge in contemporary cardiology, characterized by high morbidity and mortality rates. It poses a significant burden on healthcare systems worldwide, demanding innovative approaches to management and treatment.^[18,19]^ The intersection of CSHF with SCH adds a layer of complexity to patient prognosis. Our systematic review and meta-analysis, focusing on the question, “Does SCH Affect the Prognosis of Patients with CSHF,” offers novel insights into this intricate relationship. We found that SCH indeed influences the clinical outcomes in CSHF, signifying the need for heightened awareness and potentially tailored treatment strategies for this subgroup. This analysis not only aids in understanding the pathophysiological underpinnings linking thyroid function to heart failure but also highlights the potential for improved prognostic assessments and personalized therapeutic interventions.^[20]^ Clinically, our findings underscore the importance of screening for thyroid dysfunctions in CSHF patients and advocate for a multidisciplinary approach in managing such complex cases, ultimately aiming to enhance patient outcomes and quality of life.

The results of this meta-analysis underscore the importance of recognizing SCH as a significant risk factor for all-cause mortality in patients with heart failure. The consistency across the studies, as indicated by the low heterogeneity, strengthens the reliability of this finding. The HR of 1.42 suggests a 42% increased risk of death in heart failure patients with SCH compared to those without. This increased risk might be attributed to the subtle yet impactful effects of thyroid hormone dysregulation on cardiovascular health. SCH is known to influence cardiac contractility, systemic vascular resistance, and lipid metabolism, all of which are crucial in the pathophysiology of heart failure. Additionally, SCH might exacerbate the underlying cardiac condition, leading to a more severe disease course. Clinically, these findings highlight the need for vigilant monitoring and potential therapeutic interventions in heart failure patients with SCH.^[21]^ While the direct causal relationship and mechanisms underlying this association warrant further investigation, the current evidence suggests that screening for thyroid function could be an integral part of managing heart failure.

This meta-analysis highlights SCH as a significant risk factor for cardiac events, a finding that is particularly relevant given the high heterogeneity among the included studies. The heterogeneity might stem from variations in study populations, definitions of cardiac events, or SCH thresholds across different studies. Despite these variations, the overall risk increases of 46% is notable. The underlying mechanisms through which SCH may increase the risk of cardiac events could involve several factors. SCH is known to affect cardiac function by altering myocardial contractility, increasing vascular resistance, and potentially leading to atherosclerotic changes. These physiological alterations can exacerbate existing cardiac conditions or even precipitate new cardiac events.^[22]^ From a clinical standpoint, these findings emphasize the importance of considering thyroid function in patients at risk of cardiac events. Regular screening for thyroid abnormalities, particularly in patients with existing cardiovascular risk factors, could be crucial. Additionally, this evidence suggests that managing SCH could potentially be an avenue for reducing the incidence of cardiac events, although further research is needed to confirm this hypothesis.

The subgroup analysis reveals that the association between SCH and all-cause mortality in heart failure patients varies depending on several factors. The increased risk in Asian, European, and American populations highlights the global relevance of this association, though the regional differences in HRs suggest potential variations in genetic, environmental, or healthcare factors. The higher risk observed in studies with smaller sample sizes could be indicative of the increased variability and potential biases inherent in smaller cohorts. In contrast, larger studies provided a more stable estimate but still showed a significant association, emphasizing the broader applicability of the findings. Age emerged as a critical determinant, with younger patients (≤65 years) exhibiting a higher risk. This might suggest that the impact of SCH on mortality is more pronounced in relatively younger patients, potentially due to longer exposure or more aggressive progression of heart-related complications in the presence of SCH. The follow-up duration also played a significant role, with shorter studies showing a higher risk. This could imply that the effects of SCH on mortality are more immediate and diminish over time, or that longer studies are more likely to include participants with a stabilized form of SCH or heart failure.

In this meta-analysis, we examined the impact of SCH on all-cause mortality and cardiac events in CSHF patients. Subgroup analysis for all-cause mortality was feasible due to extensive and consistent data, revealing that SCH significantly increases mortality risks, underscoring the need for careful thyroid management in heart failure. Conversely, the analysis of cardiac events demonstrated significant heterogeneity (*P* = .023, *I*² = 64.7%), addressed through a sensitivity analysis that confirmed the robustness of our findings despite varying study populations, methodologies, and SCH definitions. This heterogeneity highlights the importance of individualized patient assessment and adherence to local clinical guidelines in interpreting these outcomes.

One significant limitation of our research is the inherent heterogeneity among the included studies, especially in terms of sample sizes, geographic locations, and definitions of SCH. This diversity could potentially impact the generalizability of our findings. Additionally, most studies in our meta-analysis were observational, which inherently limits our ability to establish causation between SCH and CSHF outcomes. Another constraint is the lack of uniformity in the follow-up durations across studies, which might affect the long-term applicability of our results. Lastly, the absence of detailed patient-level data restricted our ability to explore deeper interactions between SCH and individual patient characteristics, which could offer more personalized insights into the management of heart failure in the context of SCH. Furthermore, the absence of blinding and allocation concealment in all studies included in the meta-analysis indicates potential biases in study designs. While the overall methodological quality of the studies was high as assessed by the NOS, these methodological limitations might influence the findings’ interpretation.

## 5. Conclusions

Our study concludes that SCH is a predictive factor for all-cause mortality, cardiovascular events, and/or rehospitalization in patients with CSHF. Screening for SCH is not only crucial in assessing the prognosis of CSHF patients but may also play a significant role in improving their outcomes.

## Author contributions

**Conceptualization:** Xiao Han.

**Formal analysis:** Xiao Han.

**Methodology:** Xiao Han.

**Resources:** Xiao Han, Xiuge Wang.

**Writing – original draft:** Xiao Han.

**Supervision:** Xiuge Wang.

**Validation:** Xiuge Wang.

**Writing – review & editing:** Xiuge Wang.

## Supplementary Material



## References

[1] Baman JR Ahmad FS. Heart failure. JAMA. 2020;324:1015. 32749448 10.1001/jama.2020.13310

[2] Tayal U Prasad S Cook SA. Genetics and genomics of dilated cardiomyopathy and systolic heart failure. Genome Med. 2017;9:20. 28228157 10.1186/s13073-017-0410-8 PMC5322656

[3] Reddy YNV Borlaug BA O’Connor CM Gersh BJ. Novel approaches to the management of chronic systolic heart failure: future directions and unanswered questions. Eur Heart J. 2020;41:1764–74. 31199474 10.1093/eurheartj/ehz364

[4] Man JP Jugdutt BI. Systolic heart failure in the elderly: optimizing medical management. Heart Fail Rev. 2012;17:563–71. 22002260 10.1007/s10741-011-9282-y

[5] Biondi B. Mechanisms in endocrinology: heart failure and thyroid dysfunction. Eur J Endocrinol. 2012;167:609–18. 22956554 10.1530/EJE-12-0627

[6] AlGhalayini K. Prevalence of hypothyroidism in a cohort of Saudi women with heart failure and effect on systolic and diastolic function. J Pak Med Assoc. 2015;65:1300–4. 26627511

[7] Bielecka-Dabrowa A Godoy B Suzuki T Banach M von Haehling S. Subclinical hypothyroidism and the development of heart failure: an overview of risk and effects on cardiac function. Clin Res Cardiol. 2019;108:225–33. 30091084 10.1007/s00392-018-1340-1

[8] Page MJ McKenzie JE Bossuyt PM. The PRISMA 2020 statement: an updated guideline for reporting systematic reviews. BMJ. 2021;372:n71. 33782057 10.1136/bmj.n71 PMC8005924

[9] Wells G. The Newcastle–Ottawa Scale (NOS) for assessing the quality of non-randomised studies in meta-analyses. In: Symposium on Systematic Reviews: Beyond the Basics. Ottawa Hospital Research Institute, Ottawa, Canada. 2014; 2014.

[10] Frey A Kroiss M Berliner D. Prognostic impact of subclinical thyroid dysfunction in heart failure. Int J Cardiol. 2013;168:300–5. 23041000 10.1016/j.ijcard.2012.09.064

[11] Iacoviello M Guida P Guastamacchia E. Prognostic role of sub-clinical hypothyroidism in chronic heart failure outpatients. Curr Pharm Des. 2008;14:2686–92. 19006851 10.2174/138161208786264142

[12] Kannan L Shaw PA Morley MP. Thyroid dysfunction in heart failure and cardiovascular outcomes. Circ Heart Fail. 2018;11:e005266. 30562095 10.1161/CIRCHEARTFAILURE.118.005266 PMC6352308

[13] Li X Yang X Wang Y Ding L Wang J Hua W. The prevalence and prognostic effects of subclinical thyroid dysfunction in dilated cardiomyopathy patients: a single-center cohort study. J Card Fail. 2014;20:506–12. 24858054 10.1016/j.cardfail.2014.05.002

[14] Nanchen D Gussekloo J Westendorp RG.; PROSPER Group. Subclinical thyroid dysfunction and the risk of heart failure in older persons at high cardiovascular risk. J Clin Endocrinol Metab. 2012;97:852–61. 22238391 10.1210/jc.2011-1978

[15] Rhee CM Curhan GC Alexander EK Bhan I Brunelli SM. Subclinical hypothyroidism and survival: the effects of heart failure and race. J Clin Endocrinol Metab. 2013;98:2326–36. 23720788 10.1210/jc.2013-1039 PMC3667266

[16] Sato Y Yoshihisa A Kimishima Y. Subclinical hypothyroidism is associated with adverse prognosis in heart failure patients. Can J Cardiol. 2018;34:80–7. 29275887 10.1016/j.cjca.2017.10.021

[17] Wang W Guan H Gerdes AM Iervasi G Yang Y Tang YD. Thyroid status, cardiac function, and mortality in patients with idiopathic dilated cardiomyopathy. J Clin Endocrinol Metab. 2015;100:3210–8. 26052725 10.1210/jc.2014-4159

[18] McMurray JJ. Clinical practice. Systolic heart failure. N Engl J Med. 2010;362:228–38. 20089973 10.1056/NEJMcp0909392

[19] Botto GL Healey JS. Clinical outcomes in patients with systolic heart failure. Are atrial high rate episodes a common marker of disease progression? Int J Cardiol. 2020;316:184–5. 32380251 10.1016/j.ijcard.2020.04.077

[20] Triggiani V Angelo Giagulli V De Pergola G Licchelli B Guastamacchia E Iacoviello M. Mechanisms explaining the influence of subclinical hypothyroidism on the onset and progression of chronic heart failure. Endocr Metab Immune Disord Drug Targets. 2016;16:2–7. 26680772 10.2174/1871530316666151218151319

[21] Mitchell JE Hellkamp AS Mark DB. Thyroid function in heart failure and impact on mortality. JACC Heart Fail. 2013;1:48–55. 24159562 10.1016/j.jchf.2012.10.004 PMC3803999

[22] Abdel-Moneim A Gaber AM Gouda S Osama A Othman SI Allam G. Relationship of thyroid dysfunction with cardiovascular diseases: updated review on heart failure progression. Hormones (Athens). 2020;19:301–9. 32488814 10.1007/s42000-020-00208-8

