# Supplementary material for: Does subclinical hypothyroidism affect the prognosis of patients with chronic systolic heart failure: A systematic review and meta-analysis
Source: Medicine (Baltimore). 2024 Jun 7;103(23):e38410. doi: 10.1097/MD.0000000000038410 (PMC11155587; doi:10.1097/MD.0000000000038410)
Supplement: Supplementary file 1 [file medi-103-e38410-s001.docx]

| **Supplementary S1. Full Search Strategy Employed for Each Database** | |
| --- | --- |
| **DATABASE** | **SEARCH STRATEGY** |
| **PubMed** | **("heart failure"[Title/Abstract] OR "cardiac failure"[Title/Abstract]) AND "subclinical hypothyroidism"[Title/Abstract] AND "prognosis"[Title/Abstract]** |
| **Embase** | **('heart failure'/exp OR 'cardiac failure'/exp OR 'heart failure' OR 'cardiac failure') AND ('subclinical hypothyroidism'/exp OR 'subclinical hypothyroidism') AND ('prognosis'/exp OR 'prognosis') in [Article Title, Abstract, Keywords]** |
| **Web of Science** | **TS=((("heart failure" OR "cardiac failure") AND "subclinical hypothyroidism" AND "prognosis"))** |
| **Cochrane Library** | **("heart failure" OR "cardiac failure" OR "subclinical hypothyroidism" OR "prognosis"):ti,ab,kw** |
